# Infectious Risks Associated with Biologic Therapies in Autoimmune, Rheumatologic and Dermatologic Diseases: A Narrative Review

**DOI:** 10.3390/microorganisms14061250

**Published:** 2026-06-02

**Authors:** Stefania Capuccio, Francesco Romano, Joan R. Rello, Antonios Katsounas, Jordi Rello

**Affiliations:** 1 Department of Clinical and Experimental Medicine, University of Catania, 95122 Catania, Italy; stefania.cap@hotmail.com; 2 Department of Medicine and Surgery, “Kore” University of Enna, 94100 Enna, Italy; 3 Department of Public Health and Infectious Diseases, Sapienza University of Rome, Policlinico Umberto I of Rome, 00161 Rome, Italy; 4 Microbiology Department, Pharmacy Faculty, University of Barcelona, 08035 Barcelona, Spain; jrellori7@alumnes.ub.edu; 5 Ruhr University Bochum, Knappschaft Kliniken University Hospital Bochum, Department of Medicine, 44780 Bochum, Germany; 6 Division of Anaesthesia, Critical Care, Pain and Emergency Medicine, Nîmes University Hospital, University of Montpellier, 30900 Nîmes, France; 7 Medicine Department, Universitat Internacional de Catalunya, 08195 Barcelona, Spain; 8 Clinical Research Pneumonia and Sepsis (CRIPS) Research Group-Vall d’Hebron Institute Research (VHIR), 08035 Barcelona, Spain; 9 Centro de Investigación Biomédica en Red de Enfermedades Respiratorias (CIBERES), Instituto Salud Carlos III, 28029 Madrid, Spain

**Keywords:** autoimmune disease, infectious diseases, biological therapy, infection risk

## Abstract

Biologic and targeted synthetic therapies have substantially improved the management of autoimmune diseases (ADs), achieving unprecedented disease control. However, by modulating key immune pathways, these agents increase susceptibility to a wide spectrum of infections. This narrative review synthesizes current evidence on infectious risks associated with biologic DMARDs (bDMARDs) and targeted synthetic DMARDs (tsDMARDs) in AD, characterizing infection profiles across different drug classes, identifying patient- and treatment-related risk factors, and providing evidence-based recommendations for screening, prevention, and management. A comprehensive literature search was conducted through March 2026, across PubMed, Embase, and the Cochrane Library, using predefined search terms combining biologic and targeted synthetic drug classes with infection-related outcomes. Evidence from major international registries (BSRBR-RA, DANBIO, RABBIT) and society guidelines (ACR, EULAR, IDSA) was prioritized. Among bDMARDs, TNF-α inhibitors (TNF-α i) and rituximab were associated with the highest rates of serious infections, whereas IL-17 and IL-23 inhibitors demonstrated comparatively lower infectious risk profiles. Steroids, older age, and prior serious infections emerged as the most consistent patient-related risk modifiers. Unlike prior reviews focused on single diseases or drug classes, this work provides an integrated, cross-disciplinary risk stratification framework. bDMARDs and tsDMARDs remain among the most innovative treatments available for effective management of ADs, with favorable benefit–risk profiles when accompanied by systematic prevention strategies. Universal pre-treatment screening for tuberculosis and viral hepatitis, risk-stratified parasitic screening, evidence-based vaccination, and selective antimicrobial prophylaxis can mitigate infectious complications.

## 1. Introduction

Biologic and targeted synthetic therapies have revolutionized the treatment of numerous inflammatory dermatological and rheumatological diseases with autoimmune pathogenesis, such as psoriasis, seronegative arthropathies (SA), rheumatic arthritis (RA), inflammatory bowel diseases (IBDs), hidradenitis suppurativa (HS), atopic dermatitis, chronic urticaria, vitiligo, and many others, improving quality of life in patients with these diseases [1].

Although IBDs are primarily gastroenterological conditions, they are included in this review due to their shared immunopathogenic mechanisms with other autoimmune diseases (ADs), the substantial overlap in biologic and targeted synthetic therapies, and the frequent extra-intestinal manifestations that place IBD at the intersection of multiple specialties.

ADs are chronic inflammatory diseases with similar pathophysiology. Their most distinctive characteristic is the loss of immunological tolerance to self-antigens, which promotes an uncontrolled autoimmune response against them, causing damage to tissues, organs, or even body systems. The chronic nature of these diseases places a significant burden on health system programs, with a negative impact on patients’ quality of life [2].

There is some concern regarding the potential link between these therapies and safety risks, including infections. Some interleukins (ILs), such as tumor necrosis factor alpha (TNF-α) or IL-17, or clusters of differentiation, such as CD20, are key factors in the immune response against specific infections. Blocking them could promote infections and their progression in predisposed individuals [3].

Janus kinase inhibitors (JAKis) have recently emerged as drugs whose efficacy has been widely demonstrated across numerous diseases, both dermatological (atopic dermatitis, vitiligo, and alopecia areata) and non-dermatological (rheumatoid arthritis, myelodysplastic syndrome, and inflammatory bowel disease). Despite this, JAKis have been associated with various adverse events, such as infections, including severe ones, and an increased risk of malignancies [4].

Despite the growing body of literature on individual drug classes or single diseases, several critical knowledge gaps remain. First, comparative data on infection risk across biologic classes are largely derived from indirect comparisons. Second, the interaction between patient-specific risk factors and drug-specific immunosuppressive mechanisms remains insufficiently characterized. Third, real-world adherence to pre-treatment screening protocols and vaccination recommendations is inadequately documented, with emerging evidence suggesting significant implementation gaps between guideline recommendations and clinical practice.

This literature review aims to collect and present in a clear, precise, and comprehensive manner the currently available evidence on the impact of these drugs on the development of infectious diseases. Particular attention is paid to the underlying immunological mechanisms, the spectrum of infections observed (ranging from mild to severe), and the differences among the various biologic drugs and targeted therapies currently in use, to identify knowledge gaps that, once addressed, could help reduce infection-related complications associated with these therapies.

The goal is to provide clinicians with an updated and comprehensive overview to support risk assessment in these patients and guide informed therapeutic decisions, as well as to highlight preventive measures aimed at minimizing infection-related complications associated with the use of these drugs.

## 2. Methods

We performed literature searches across PubMed/MEDLINE, Embase, Cochrane Library, and Web of Science to identify relevant studies evaluating the association between biologic therapies and the risk of infections in AD, covering publications through March 2026. The search strategy combined MeSH and free-text terms organized into three blocks: (1) biologic therapies and targeted synthetic DMARDs, interleukin inhibitors, B-cell depleting agents, co-stimulation modulators, and JAKis; (2) infections (including opportunistic, bacterial, viral, fungal, mycobacterial, and parasitic); (3) AD (including dermatologic, rheumatologic, and inflammatory bowel diseases). Eligible study designs included randomized controlled trials, observational studies, registry-based analyses, systematic reviews, meta-analyses, and clinical practice guidelines from major professional societies (ACR, EULAR, AAD, IDSA).

Given the heterogeneity of study designs, patient populations, outcome definitions, and follow-up durations across included studies, a formal meta-analysis was not feasible; therefore, findings were synthesized qualitatively using a narrative approach. Findings were organized according to drug class (TNF-αi, IL-6 inhibitors, IL-17/IL-23 inhibitors, B-cell inhibitors, and JAKi) and by infection type (bacterial, viral, fungal, mycobacterial, and parasitic).

Particular emphasis was placed on elucidating the immunological mechanisms by which each drug class predisposes to specific infections, characterizing clinically relevant and potentially life-threatening infectious complications, and formulating practical evidence-based recommendations for pre-treatment screening, vaccination, antimicrobial prophylaxis, and ongoing monitoring strategies.

## 3. Overview of bDMARDs and tsDMARDs

Agents used to treat autoimmune and inflammatory diseases are collectively referred to as disease-modifying antirheumatic drugs (DMARDs). These are classified into three main categories: conventional synthetic DMARDs (csDMARDs), such as methotrexate and sulfasalazine; biologic DMARDs (bDMARDs); and targeted synthetic DMARDs (tsDMARDs). This review focuses on bDMARDs and tsDMARDs, which are described below [5,6].

bDMARDs are defined as engineered proteins, most commonly monoclonal antibodies or fusion proteins, that specifically target and modulate key molecules or cells involved in the dysregulated immune response characteristic of autoimmune disorders. These agents are designed to interfere with critical steps in inflammation and immune activation, such as blocking cytokines (e.g., TNF-α, IL-6, IL-17, IL-23), depleting B cells, or inhibiting T-cell co-stimulation, thus reducing pathological immune activity while minimizing generalized immunosuppression (Table 1) [7,8]. These drugs have revolutionized the treatment of AD, offering a superior mechanism of action to traditional immunosuppressive therapies, mainly due to their high target specificity, a more favorable safety profile, and greater efficacy in many refractory conditions [9].

tsDMARDs are small molecules that inhibit intracellular signaling pathways. The most prominent examples are JAKis, which block the JAK-STAT signaling cascade. Although tsDMARDs share therapeutic indications with bDMARDs in many ADs, they differ in their mechanism of action: while bDMARDs target extracellular cytokines or cell-surface receptors, tsDMARDs act intracellularly. For simplicity, the term “biologic therapies” may occasionally be used in this review when referring collectively to both bDMARDs and tsDMARDs, although the distinction between these two classes will be maintained wherever it is clinically relevant [10,11].

Despite the numerous advantages of these drugs, there are some complications and adverse effects that need to be recognized and thoroughly investigated. bDMARDs and tsDMARDs, by modulating key immune pathways, can impair host defense mechanisms, resulting in secondary immunodeficiency. The specific infection risks associated with each drug class are discussed in detail in Section 4.

Some bDMARDs, such as IL-17 inhibitors, are associated with mucocutaneous candidiasis and may exacerbate or trigger inflammatory bowel disease [12]. Another important issue is that bDMARDs may also induce paradoxical reactions and drug-induced autoimmunity, including lupus-like syndromes, paradoxical psoriasis, vasculitis, and neuropathies. These effects are more frequently reported with TNF-α i [13]. Furthermore, despite the expanding therapeutic landscape, important challenges remain, including drug immunogenicity, primary and secondary non-response, and high treatment costs, which have driven the development of biosimilars and novel drug delivery systems aimed at optimizing therapeutic outcomes and broadening patient access [14]. Finally, for several bDMARDs and tsDMARDs, especially newer agents, robust long-term safety data are still lacking, particularly regarding rare or delayed adverse events [8,15].

**Table 1:** Infection risks associated with bDMARD and tsDMARD therapies in AD.

Drug Class	Examples	Mechanism/Target	Infection Risk	Special Considerations	References
TNF-αi	Infliximab, Adalimumab, Certolizumab pegol, Etanercept	Neutralize TNF-α Reduce inflammation	🔴 TB, HBV, opportunistic, granulomatous infections	Screen for latent TB; monitor viral reactivation	Baddley et al., (2018) [16]; Lv et al., (2026) [17]
IL-6i	Tocilizumab	IL-6 receptor blockade	🟡 Skin/respiratory infections, oral/esophageal candidiasis, VZV, M. tuberculosis, atypical mycobacteria, histoplasmosis	Careful screening; vaccination before therapy	Campbell et al., (2021) [18]; Bellan et al. (2022) [19]
IL-17 IL-23i	Secukinumab, Ixekizumab, Brodalumab, Bimekizumab, Guselkumab, Tildrakizumab, Risankizumab	Block Th17 (IL-17) or IL-23p19	🟢 Nasopharyngitis, upper respiratory infections, susceptibility to M. tuberculosis	Monitor respiratory and mucocutaneous infections	Wu et al. (2023) [20]; Blauvelt et al., (2025) [21]; Kridin et al. (2023) [22]
B-cell Targeted	Rituximab, Ofatumumab, Ocrelizumab, Ublituximab, Belimumab, Obinutuzumab, Anti-BAFF/APRIL	Anti-CD20/CD19/CD22 BTK inhibition BAFF/APRIL blockade	🟡 Opportunistic infections (VZV, CMV, HBV, PCP, PML)	HBV screening; PCP prophylaxis in high-risk patients; risk ↑ with hypogammaglobulinemia and comorbidities	Perrone et al. (2026) [23]; Sisi et al. (2025) [24]
JAKi	Tofacitinib, Baricitinib, Upadacitinib	Inhibit JAK/STAT signaling Modulate cytokine responses	🟢 Pneumonia, nasopharyngitis, UTI, cellulitis, VZV; TB risk	Screen for latent TB; monitor for VZV reactivation; low opportunistic infection	Olivera et al. (2020) [25]; Laskou et al., (2026) [26]

Abbreviations: TB, Tuberculosis; HBV, Hepatitis B Virus; CMV, Cytomegalovirus; PML, Progressive Multifocal Leukoencephalopathy; PCP, *Pneumocystis jirovecii* Pneumonia; VZV, Varicella Zoster Virus; UTI, Urinary Tract Infection; JAK, Janus Kinase; BTK, Bruton’s Tyrosine Kinase; BAFF, B-cell Activating Factor; APRIL, A Proliferation-Inducing Ligand; TNF-αi, TNF-α Inhibitors; IL-6i, IL-6 Inhibitors; IL-17 IL-23i, IL-17 IL-23 Inhibitors; JAKi, JAK Inhibitors. Legend (Infection Risk): 🟢 low/mild; 🟡 moderate/occasionally serious; 🔴 high/frequent or severe.

### 3.1. TNF-α Inhibitors

TNF-α is a pleiotropic primary cytokine of the innate immune system. This cytokine is involved in the pathophysiology of many inflammatory disorders, RA, seronegative spondyloarthropathies, and inflammatory bowel disease. TNF-α is mainly produced by macrophages and T-lymphocytes and is initially synthesized as a membrane-bound precursor, then cleaved by the TNF-α-converting enzyme (TACE), which releases a soluble circulating form. TNF-α exerts its effects by binding to TNF receptor 1 or TNF receptor 2, triggering a broad inflammatory response. This activation promotes the production of other pro-inflammatory cytokines (such as IL-1β, IL-6, and IL-8), chemokines, and adhesion molecules, ultimately enhancing macrophage and neutrophil recruitment and function [27,28]. Overall, TNF-α plays a key role in chemotaxis, phagocytosis, and in the formation and maintenance of granulomas [29]. The TNFα antagonist group includes infliximab, adalimumab, and certolizumab pegol, which are monoclonal antibodies against TNFα, and etanercept, a TNFα receptor fusion protein, binding to soluble TNFα [12]. These medications are the longest-used biologic agents in the treatment of psoriasis; they are also widely used in the treatment of other diseases, such as psoriatic (PsA) and other SA, RA, IBD, HS, and uveitis [30].

### 3.2. IL-6 Inhibitors

Interleukin 6 (IL-6) is a secreted protein that consists of 184 amino acids and two N-glycosylation sites as well as four cysteine residues. IL-6 is a multifunctional cytokine that acts across various biological systems and organs. It is an essential cytokine that transmits defense signals from sites of pathogen invasion or tissue damage, stimulating acute-phase responses, immune reactions, hematopoiesis, and multiple internal organs to prepare for host defense [31]. Macrophages and dendritic cells produce IL-6 after toll-like receptors (TLRs) detect pathogens at the site of infection or tissue injury. IL-6 has pleiotropic biological effects on immunological responses, acute-phase responses, and hematopoiesis [32]. Since IL-6 is involved in several disease processes, its inhibition has been suggested as a possible way to improve clinical outcomes. The development of strategies aimed at inhibiting IL-6 began with the introduction of the anti-IL-6 receptor monoclonal antibody tocilizumab (TCZ) [33], which has been approved in more than 100 countries for the treatment of patients with autoimmune disorders [34].

Clinical studies of IL-6 inhibitors, mainly tocilizumab, reveal that their use is associated with an increased rate of both serious and opportunistic infections, generally in the range observed with other non-IL-6-directed biologic therapies [35].

### 3.3. IL-17 and IL-23 Inhibitors

IL-17 is essential in the host defense against mucocutaneous candidiasis, inducing neutrophil chemotaxis and antimicrobial peptide activity [36]. Patients with inborn defects in IL-17 immunity display severe and chronic skin and mucosal candidiasis [37]. Biologics inhibiting the T-helper (Th)-17 pathway (secukinumab, ixekizumab, brodalumab, bimekizumab) have been approved for moderate-to-severe plaque psoriasis in adults and children, active psoriatic arthritis in adults, active ankylosing spondylitis, non-radiographic axial spondyloarthritis with objective signs of inflammation, enthesitis-related arthritis in pediatric patients, and moderate-to-severe HS in adults [38].

IL-23 is a heterodimeric cytokine composed of two protein subunits, p40 and p19. The p40 subunit is shared with IL-12, whereas p19 is unique to IL-23. Inhibitors targeting the p19 subunit represent important therapeutic options for patients with psoriasis and include Guselkumab, Tildrakizumab, and Risankizumab. Similar to IL-17A, IL-23 has also been implicated in protective immune responses against infection caused by *Mycobacterium tuberculosis* [39].

### 3.4. B-Cell Inhibitors

B cells play a central role in the immune system, contributing to antibody production, the establishment of immunological memory, and the regulation of immune responses. Dysregulated B cell activation is strongly linked to autoantibody generation, which can target healthy tissues and drive systemic inflammation and organ damage [40]. The advent of B-cell-directed biologic therapies marks a significant milestone in the management of AD [41].

B-cell-targeted therapies exert their effects through diverse mechanisms, including the engagement of surface markers such as CD19, CD20, and CD22, blockade of B-cell survival factors via anti-BAFF or anti-APRIL antibodies, and inhibition of Bruton’s tyrosine kinase (BTK). This mechanistic heterogeneity implies that the efficacy and safety profiles of different B-cell-directed agents may differ substantially [42]. Moreover, many of these agents are associated with neutropenia, including both early- and late-onset neutropenia, which can contribute to the risk of infections. There are currently five anti-CD20 monoclonal antibodies licensed for the treatment of B-cell malignancies and/or autoantibody-mediated AD: rituximab, ofatumumab, ocrelizumab, obinutuzumab, and ublituximab [43,44].

Belimumab, as a human immunoglobulin G1λ monoclonal antibody (mAb) targeting B lymphocyte stimulator, was approved by the US Food and Drug Administration (FDA) and European Medicine Agency (EMA) in 2011 for the treatment of systemic lupus erythematosus (SLE) [45].

Prospective studies and real-world analyses indicate that the risk of infections with these agents is comparable to rituximab and standard therapy, without a significant increase in serious infections. BAFF inhibitors are a class of innovative biologic drugs that act by blocking the B-cell survival signal, which is crucial in the pathogenesis of many ADs [46]. BAFF is a TNF family cytokine that promotes B-cell maturation, survival, and differentiation, thereby favoring the persistence of autoreactive B cells and the production of autoantibodies. These drugs work either by neutralizing circulating BAFF (e.g., belimumab) or by blocking the BAFF receptor (BAFF-R) (e.g., ianalumab), ultimately leading to reduced survival of autoreactive B cells [47].

The currently recognized clinical indications approved by the FDA in the United States are active SLE, in combination with standard therapy, in both adults and children aged ≥5 years, and active lupus nephritis, also in combination with standard therapy [48,49].

### 3.5. JAK Inhibitors

The development of small molecule inhibitors of Janus kinases (JAKis), classified as tsDMARDs, has offered an alternative to bDMARDs in immune-mediated inflammatory diseases. Demonstrating impressive efficacy in treating conditions mediated by the JAK/signal transducers and activators of transcription (STAT) pathways, such as RA, psoriatic arthritis, ulcerative colitis, and psoriasis. Janus kinases are members of the tyrosine kinase family that play a key role in transferring extracellular signals into the nucleus, altering DNA transcription, downstream translation, and effector protein manufacture [50].

Cell surface receptors require a pair of JAKs as either identical homodimers (e.g., JAK2/JAK2) or heterodimers (e.g., JAK1/JAK3) in order to signal. This, in turn, activates STAT proteins, which target gene promoters to activate transcription. The JAK/STAT pathways downregulate more than 50 cytokines and growth factors and are considered a fundamental communication node for the immune system [51].

Human studies on JAK-STAT signaling have shown that germline loss- or gain-of-function mutations in JAK or STAT genes lead to diverse immunological disorders and increased infection susceptibility. For instance, JAK3 loss-of-function causes severe combined immunodeficiency (SCID), while mutations in JAK1, TYK2, STAT1, and STAT5B are linked to intracellular bacterial infections. STAT5B deficiency interferes with normal T-cell differentiation and memory development, thereby predisposing affected individuals to recurrent pneumonia. STAT1 loss-of-function interferes with type I and II interferon responses, increasing viral susceptibility, whereas STAT1 gain-of-function mutations are associated with recurrent *Candida* infections by antagonizing STAT3-mediated antifungal immunity [52,53].

## 4. Types of Infections

Evidence from patients with ADs, including RA, psoriasis, vitiligo, inflammatory bowel diseases, and SLE, indicates that bacterial infections, particularly of the respiratory and urinary tracts, viral infections such as herpes zoster, herpes simplex, hepatitis B, and cytomegalovirus, fungal infections including candidiasis, aspergillosis, and pneumocystis, as well as parasitic infections like leishmaniasis and strongyloidiasis, represent the most clinically relevant infectious complications [54,55,56] (Table 2).

The incidence and spectrum of infections change according to both the biologic’s mechanism of action and the underlying disease. TNF-α inhibitors are associated with an increased risk of granulomatous infections such as TB and listeriosis, bacterial infections including pneumonia and sepsis, viral infections such as herpes zoster, and fungal infections including candidiasis and aspergilosis [57]. IL-17 inhibitors predominantly increase susceptibility to mucocutaneous candidiasis and respiratory infections, whereas JAKis are linked to a higher risk of herpes zoster and opportunistic infections [55]. Anti-CD20 therapies, including rituximab, have been associated with severe bacterial infections, hepatitis B reactivation, and viral infections [58], while IL-12/23 and IL-23 inhibitors generally carry a lower overall infection risk but may predispose patients to respiratory and urinary tract infections [55]. Mild infections, such as those affecting the upper respiratory tract, are very common and can significantly impact quality of life [59]. Severe infections are more common during the early months of therapy, in elderly patients, in those with comorbidities, or when corticosteroids are used concomitantly [1].

Preventive strategies, including screening for latent TB and hepatitis B/C, vaccination, and close clinical monitoring, remain essential in patients receiving bDMARDs and tsDMARDs [1,60].

**Table 2:** Overview of infection risks associated with biologic therapies in AD.

Infection Type	Main Biologics	Manifestations	Prevention Management	References
Bacterial	TNF-αi, Tocilizumab, Rituximab, Abatacept, JAKi	Pneumonia, sepsis, UTI (*E. coli*, *K. pneumoniae*), cellulitis, erysipelas, necrotizing fasciitis, cutaneous abscesses (*S. aureus*/*MRSA*)	Pneumococcal and influenza vaccination pre-treatment; regular clinical monitoring	Li et al. (2021) [61]; Leding et al. (2026) [62]; Jiang et al. (2024) [63]
Viral	JAKi TNF-αi	Reactivation of latent virus	Vaccination, antivirals, clinical monitoring	Lan et al. (2025) [64]; Shin et al. (2026) [65]
Fungal	TNF-αi Rituximab Tocilizumab JAKi	Candidiasis, histoplasmosis, aspergillosis, dermatophytosis	Clinical monitoring, early management of infections	Barbosa et al. (2025) [66]; Malpica et al. (2019) [67]
Mycobacterial	TNF-αi Rituximab Abatacept, JAKi	TB, NTM, atypical presentations	Latent TB screening, prophylaxis, clinical monitoring	Picchianti-Diamanti et al. (2025) [68]
Parasitic	TNF-αi Anti-IL-1/6, Anti-CD20 CTLA4-Ig	Strongyloides, Toxoplasma, Leishmania, Chagas; severe/disseminated forms	Pre-treatment screening, targeted prophylaxis, clinical monitoring	Lo et al. (2025) [69]; Ciudad et al. (2026) [70]

Abbreviations: JAKi, Janus kinase inhibitors; TNF-αi, tumor necrosis factor α inhibitors; CTLA4-Ig, cytotoxic T-lymphocyte-associated protein 4 immunoglobulin fusion protein; TB, tuberculosis; NTM, nontuberculous mycobacteria.

### 4.1. Bacterial Infection

TNF-α inhibitors reduce phagocytic function and heighten the risk of granulomatous infection. However, there is also an increased risk of invasive viral and parasitic disease [71]. TNF-α is an important cytokine against viral disease; indeed, it promotes hepatitis B virus-specific cytotoxic T-lymphocyte [72]. This cytokine plays a significant role in the immune defense against Cryptococcus neoformans, pulmonary histoplasmosis, and *Candida albicans*. TNF-α inhibitors also increase the risk of invasive viral and parasitic disease [73]. Another well-known side effect of anti-TNFα drugs is an increased risk of TB development [74]. Bacterial infections are among the most frequently reported infections in patients treated with bDMARDs and tsDMARDs. The most severe bacterial infections associated with the use of these drugs for the treatment of AD include bacterial pneumonia, sepsis, complicated urinary tract infections, skin and soft tissue infections (including cellulitis and necrotizing fasciitis), and active TB [75]. These infections may lead to hospitalization, the need for intravenous antibiotic therapy, and, in the most severe cases, death [76]. The risk of serious infections is considerably increased, particularly with TNF inhibitors, with a particularly high risk of active TB, especially when appropriate screening and prophylaxis are not performed [57]. Tocilizumab is associated with a higher risk of serious infections compared with anti-TNF agents, particularly pneumonia and sepsis. Rituximab and abatacept show a risk comparable to anti-TNF agents; however, rituximab has been associated with a higher risk of respiratory infections and sepsis [77]. Thirty-day mortality after a serious infection may exceed 10% in patients treated with bDMARDs and tsDMARDs [75].

The severity of infections also depends on the presence of comorbidities, concomitant use of corticosteroids and immunosuppressants, and the duration of biologic therapy [78]. Prevention of significant infections requires TB screening, vaccinations (influenza, pneumococcal), and close clinical monitoring, as recommended by the British Society for Rheumatology [79]. Infections associated with biologic drugs do not occur in all patients; however, the risk is significantly increased compared with the general population. Several specific risk factors increase the likelihood of infection, including older age, female sex, comorbidities (diabetes, renal impairment, chronic lung disease), and high doses or combinations of biologic therapies [80].

The risk also varies according to the type of agent. Anti-TNF therapies (particularly infliximab) are associated with a higher risk of serious infections, including active TB, bacterial pneumonia, sepsis, and opportunistic infections such as *Pneumocystis jirovecii* pneumonia and herpes zoster [81]. JAKis increase the risk of significant infections and herpes zoster, with a dose-dependent effect. Bacterial pneumonia may occur at any time after initiation of biologic therapy; however, the risk is particularly high during the first 6–12 months of treatment, especially in patients with risk factors [80].

The most common causative pathogens are *Streptococcus pneumoniae* and *Haemophilus influenzae*; in immunosuppressed patients, *Staphylococcus aureus* and *Pseudomonas aeruginosa*, and, in selected cases, *Legionella pneumophila*, particularly in patients receiving infliximab and adalimumab [56,80]. Prevention strategies include pneumococcal and influenza vaccination before initiating therapy and periodically during treatment, screening for and management of comorbidities, minimizing corticosteroid doses and using concomitant immunosuppressive agents cautiously, patient education on early recognition of infectious symptoms, and regular clinical monitoring to allow early identification of signs of infection [60,77,80]. Effective prevention and vigilant monitoring are key to reducing morbidity and mortality from bacterial pneumonia. Some bacterial infections may involve the skin and mainly include cellulitis, erysipelas, cutaneous abscesses, necrotizing fasciitis, and impetigo [82]. These infections can be caused by *Streptococcus pyogenes* and *Staphylococcus aureus*, including methicillin-resistant strains (MRSA) [56,83].

The risk of severe skin infections that require hospitalization and intravenous antibiotic therapy is increased chiefly with anti-TNF agents (especially infliximab), but it is also reported with other bDMARDs such as rituximab and tocilizumab. Risk factors include concomitant corticosteroid use, high biologic doses, comorbidities, smoking, and active rheumatologic disease [82]. It is important to note that urinary tract infections, particularly mild infections, are common, especially during the first months of therapy, and they can progress to complicated forms in immunosuppressed patients or following kidney transplantation. The main pathogens are *Escherichia coli*, followed by other enteric bacteria such as *Klebsiella pneumoniae* and *Proteus mirabilis*, and less frequently *Staphylococcus aureus* (especially in patients with catheters or urinary tract abnormalities) [84].

Anti-CD20 therapies are generally considered safe; however, serious infections, including opportunistic infections, have been reported even with monotherapy. Randomized controlled trials in various AD often do not show an increased risk of overall or serious infections compared with placebo. Nevertheless, anti-CD20 agents are relatively contraindicated in patients with severe lymphopenia and markedly reduced CD4 or CD8 T-cell counts. Another agent is ocrelizumab: in patients with multiple sclerosis, the risk of infections, particularly respiratory and serious infections, is increased compared with the general population, and is associated with hypogammaglobulinemia and treatment duration [85]. The risk of infections varies among different B-cell-targeted biologics, with a comparable profile observed for rituximab, belimumab, ocrelizumab, ofatumumab, and ublituximab. Obinutuzumab and anti-BAFF/APRIL agents appear to be associated with a slightly higher risk of overall infections, but not of serious infections [54]. The infections most frequently associated with BAFF inhibitors include upper (e.g., sinusitis, pharyngitis, bronchitis) and lower respiratory tract infections (pneumonia), which represent the most common category [48]. Urinary tract infections have also been reported. Infections caused by *Staphylococcus aureus* and other bacterial pathogens have been reported, particularly in patients with SLE receiving combination immunosuppressive therapy [49]. The literature highlights that the risk of serious infections with belimumab is generally low but may increase when combined with corticosteroids or other immunosuppressive agents.

### 4.2. Viral Infection

Viral infections associated with the use of bDMARDs and tsDMARDs mainly include reactivation of latent viruses and an increased risk of acute infections. Among all viral infections, herpes zoster stands out. The risk is particularly increased with JAKi and, to a lesser extent, with anti-TNF agents and other bDMARDs. Reactivation is associated with inhibition of innate interferon pathways [86].

Risk factors include older age, female sex, comorbidities, concomitant use of corticosteroids (particularly >15 mg/day of prednisolone equivalent), immunosuppressive agents, SLE, Behçet’s disease, and specific biologics such as infliximab and JAKi [87]. Studies indicate that the risk of zoster with JAKi is nearly double that of TNF inhibitors in certain populations [88,89].

The typical clinical presentation consists of neuropathic pain followed by a unilateral vesicular rash along the affected dermatome [90,91]. In immunosuppressed patients, disseminated forms, visceral involvement, and complications such as postherpetic neuralgia may occur. Management includes early antiviral therapy (acyclovir, valacyclovir, or famciclovir) for at least seven days, with dosing adjusted for renal function [87]. In severe or disseminated cases, intravenous administration of antiviral therapy, and temporary suspension of the bDMARD or tsDMARD may be considered, but decisions should be individualized [92].

Prevention strategies include vaccination against herpes zoster. The recombinant vaccine (Shingrix) is preferred and approved by the FDA in the USA for adults ≥ 50 years and can also be administered to immunosuppressed patients, including those on bDMARD or tsDMARD therapy. Vaccination should ideally be performed before starting immunosuppressive therapy, but it may also be considered during treatment after evaluating the patient’s risk profile [91]. The live-attenuated vaccine (Zostavax) is not recommended for immunocompromised patients [93].

Documented serious infections include primary and reactivated herpes zoster, hepatitis B and C virus reactivation, cytomegalovirus reactivation, enterovirus infections, and progressive multifocal leukoencephalopathy (PML) [94,95]. Hepatitis B reactivation has been reported in patients treated with rituximab and other anti-CD20 agents, and can progress to fulminant hepatitis with fatal outcome [96]. Therefore, all patients considered for anti-CD20 therapy should undergo hepatitis B virus screening before treatment initiation. Reactivation of Varicella zoster virus (VZV) has been described, with a slightly increased risk compared with the general population. Other viral infections (e.g., influenza, respiratory syncytial virus) have also been observed. The most frequent serious infections associated with JAKis include pneumonia, nasopharyngitis, urinary tract infections, cellulitis, and herpes zoster [97]. VZV reactivation, herpes zoster or ‘shingles’, is now the most recognized infectious complication with JAKis [98].

### 4.3. Fungal Infection

Fungal infections are well documented in the literature. In general, bDMARDs and tsDMARDs, particularly TNF-α inhibitors, increase the risk of invasive and superficial fungal infections, including histoplasmosis, candidiasis, aspergillosis, coccidioidomycosis, cryptococcosis, dermatophytosis, onychomycosis, and pityriasis versicolor [66,99]. A study conducted by Hennessee et al. has shown that the incidence of invasive fungal infections is low (<0.5% in the year following initiation of TNF-α therapy), but the risk is significantly higher compared to the general population, particularly for histoplasmosis and candidiasis [100].

Also in this case, the risk varies depending on the type of agent: infliximab, adalimumab, and etanercept are associated with a higher risk of invasive fungal infections, whereas others, such as rituximab, tocilizumab, baricitinib, and upadacitinib, show an increased risk of pulmonary fungal infections, according to FAERS data [99]. Fungal infections may present with atypical clinical manifestations and require intensive patient monitoring [101].

Clinical studies of IL-6 inhibitors, mainly tocilizumab, reveal that their use is associated with an increased rate of both serious and opportunistic infections, generally in the range observed with other non-IL-6-directed biologic therapies [102]. A meta-analysis conducted by Bilal et al. reported that the most frequent opportunistic infections associated with the use of interleukin inhibitors include oral candidiasis, esophageal candidiasis, and unspecified candidiasis [103].

Opportunistic infections are rare in patients treated with JAKis. Pooled trial and long-term extension data show low incidence rates, with Candida being the most frequent pathogen. While prophylaxis is generally not required, clinicians should remain vigilant. Careful assessment of patient-specific infection risk, prior treatments, and disease activity is essential when evaluating the potential risks and benefits of JAKis. With these considerations in mind, judicious use of JAKis can offer a transformative treatment option, particularly for patients who do not tolerate or respond to other therapies [104,105].

Prophylaxis for *Pneumocystis jirovecii* pneumonia is recommended in patients with ANCA-associated vasculitis or pemphigus vulgaris receiving anti-CD20 therapy. Infection risk appears to be associated with hypogammaglobulinemia and the presence of comorbidities [106].

Skin and respiratory infections are among the most commonly reported adverse events associated with IL-6 inhibitors. Therefore, careful evaluation of these organ systems, along with appropriate patient counseling and thorough screening and monitoring, is essential to reduce the risk of complications [102].

A meta-analysis conducted by Wu et al. showed that nasopharyngitis and upper respiratory tract infections are the most frequent adverse events observed with both IL-17 and IL-23 inhibitors [20].

The literature highlights the importance of regular monitoring, assessment of individual risk factors, and preventive strategies in patients treated with bDMARDs and tsDMARDs [107].

### 4.4. Mycobacterial Infection

Articles discussing mycobacterial infections associated with the use of bDMARDs and tsDMARDs in patients treated for AD highlight that the risk of TB and NTM infections is significantly increased in patients treated with TNF-α inhibitors compared to the general population and to patients treated with other agents [68]. The risk is particularly high with infliximab and adalimumab, whereas etanercept presents a lower risk, although still higher than in biologic-naïve patients [108].

The literature emphasizes that most cases of TB in patients with RA occur in treatment-naïve patients; however, a prospective population-based national cohort study carried out by Arkema et al. has demonstrated that exposure to bDMARDs and tsDMARDs, particularly anti-TNF agents, further increases the risk, with a hazard ratio that can reach up to 7.9 in the first years of use [109]. Other agents such as rituximab, abatacept, tocilizumab, and JAKis are also associated with TB risk, although lower compared to anti-TNF agents [85,86].

Like biologics, there has been concern about TB risk with JAKis. As a result, patients are routinely screened for latent TB before starting therapy. Phase II and III trials, as well as real-world studies, report very low TB incidence among JAKi-treated patients.

A large meta-analysis of tofacitinib, baricitinib, and upadacitinib trials found only one TB case, in a patient who had not been properly screened. Long-term extension studies confirmed low incidence rates, particularly in regions with low or medium TB prevalence, though higher doses (e.g., tofacitinib 10 mg BID, baricitinib 4 mg) and residence in endemic areas were associated with increased risk. Overall, TB risk depends on regional prevalence, but screening for latent infection remains essential due to its low cost and potential impact [104].

Infections caused by nontuberculous mycobacteria have also been reported, especially in patients with pre-existing lung damage, and they may present with unusual clinical symptoms, which can often delay diagnosis [110].

All studies agree on the need for latent TB screening before initiating bDMARD or tsDMARD therapy and on the importance of prophylaxis in high-risk individuals.

### 4.5. Parasitic Infection

The clinical implications of parasitic diseases in patients with AD treated with bDMARDs or tsDMARDs are significant due to the increased risk of infection, possible reactivation of latent parasitoses, and atypical clinical presentation. Drug-induced immunosuppression, particularly with anti-TNF, anti-IL-1, anti-IL-6, anti-CD20, and CTLA4-Ig agents, impairs cell-mediated immune responses, increasing susceptibility to intracellular parasites such as *Toxoplasma gondii*, *Strongyloides stercoralis*, *Leishmania* spp., and other protozoa and helminths [111].

Parasitic infections may be more severe, with a risk of severe complications (e.g., hyperinfection syndrome in strongyloidiasis, cerebral toxoplasmosis, and visceral leishmaniasis). Atypical clinical presentation and a weaker inflammatory response can lead to delays in diagnosis. Pre-treatment screening for latent parasitic infections is essential, especially in patients from endemic areas or with a history of travel to high-risk regions [112].

Clinical management requires a high level of suspicion, regular monitoring, and, in cases of active infection, temporary discontinuation of the biologic drug and administration of specific antiparasitic therapy [113]. Strongyloidiasis represents one of the most significant parasitic threats in patients receiving biologic therapy. Screening for *Strongyloides stercoralis* should be performed before initiating immunosuppressive treatment, particularly in patients originating from endemic areas (tropical and subtropical regions, as well as rural areas of the United States and Europe) or in those with a history of travel to high-risk countries. Serological testing for *S. stercoralis* IgG antibodies is the most commonly used screening method [69,114,115]. However, stool examination using the Baermann technique or agar plate culture may also be employed as an alternative diagnostic approach. Of particular relevance is Strongyloides hyperinfection syndrome, a severe complication associated with mortality rates exceeding 60%. It may occur long after the initial infection, once immunosuppressive therapy is initiated. The syndrome is characterized by massive parasite proliferation, gastrointestinal and pulmonary involvement, and, frequently, Gram-negative bacteremia or meningitis resulting from disruption of the intestinal barrier. Ivermectin is highly effective and should be administered prophylactically to seropositive patients before starting biologic therapy [69,116].

Toxoplasmosis is a potential concern among parasitic infections, especially in seropositive individuals. Although screening protocols are well defined for stem cell transplant recipients, data on rheumatologic patients treated with biologics are still scarce [117]. In transplant populations, trimethoprim–sulfamethoxazole (TMP-SMX) provides effective prophylaxis against both *Toxoplasma gondii* and *Pneumocystis jirovecii*. Monitoring with quantitative PCR in blood can detect infection before the onset of clinical disease [118]. In patients treated with biologics, routine screening is not universally recommended; however, serologic testing may be considered in individuals from endemic areas or in those receiving intensive immunosuppression. In immunocompromised patients, the most common clinical manifestations include encephalitis and pneumonitis [117,118].

Regarding leishmaniasis, current guidelines do not recommend routine serologic screening in asymptomatic individuals before initiating biologic therapy, even in the presence of previous exposure in endemic regions. Nevertheless, patients with documented asymptomatic infection or a history of visceral leishmaniasis should nonetheless undergo vigilant clinical monitoring. If visceral disease develops, the treatment of choice is liposomal amphotericin B, and immunosuppressive therapy should be reduced whenever possible. In cases of cutaneous leishmaniasis associated with the use of TNF-α antagonists, systemic treatment is recommended, and discontinuation of the biologic agent should be considered [119].

Chagas disease, caused by *Trypanosoma cruzi*, carries a significant risk of reactivation in immunosuppressed patients. Screening is recommended for individuals who have lived for at least six months in endemic areas of Latin America before starting immunosuppressive therapy. Reactivation occurs in approximately 27% of immunosuppressed patients, with higher rates observed in transplant recipients and lower rates in patients with AD. Monitoring with quantitative PCR allows early detection of reactivation and the prompt initiation of preventive treatment with benznidazole. Among immunosuppressive agents, mycophenolate mofetil appears to be associated with a particularly high risk, likely due to its effects on CD8+ T-cell depletion [120,121].

Primary prevention of parasitic infections in patients receiving biologic therapies should first and foremost rely on patient education aimed at reducing exposure risk. Patients should be advised on food and water safety, particularly when traveling to endemic areas, and on avoiding behaviors that may increase the risk of infection. Regarding toxoplasmosis, recommendations include appropriate food handling, avoiding undercooked meat, and taking precautions when handling cat litter [117,119].

Risk stratification should guide the intensity of screening and monitoring. Patients at higher risk include those who come from or have lived for prolonged periods in endemic areas, individuals with a history of parasitic infections, and patients receiving intensive immunosuppression, such as combination therapy or high-dose corticosteroids. The type of biologic agent also influences infection risk: anti-TNF agents, anti-CD20 therapies, and JAKis have been associated with higher infection rates compared with some other biologic treatments [122,123].

Finally, close multidisciplinary collaboration between rheumatologists, infectious disease specialists, and experts in tropical medicine is essential for the optimal management of these patients [60]. The adoption of structured protocols for screening, monitoring, and treatment can help reduce the morbidity and mortality associated with these preventable complications [124].

### 4.6. Critical Appraisal of the Evidence

It is important to acknowledge several limitations in the current evidence base. Most comparative data on infection risk across bDMARD and tsDMARD classes are derived from indirect comparisons through network meta-analyses, as head-to-head trials with infection as a primary endpoint are lacking. Furthermore, clinical trial populations may not fully reflect real-world patients, who are often older, have more comorbidities, and receive concomitant immunosuppressive agents [26,125]. Contradictory findings exist regarding the relative infection risk of certain agents: for example, while some registry data suggest tocilizumab carries a higher infection risk than TNF-α inhibitors, randomized controlled trials have not consistently confirmed this difference, likely due to differences in patient selection and follow-up duration [126]. Similarly, the infection risk attributed to JAKis versus bDMARDs remains debated, with the ORAL Surveillance trial raising safety concerns for tofacitinib that may not be generalizable to all JAKis or all patient populations [127]. These discrepancies underscore the need for prospective, head-to-head comparative studies with standardized infection endpoints across drug classes.

## 5. Risk Factors for Infections

**Figure 1:**
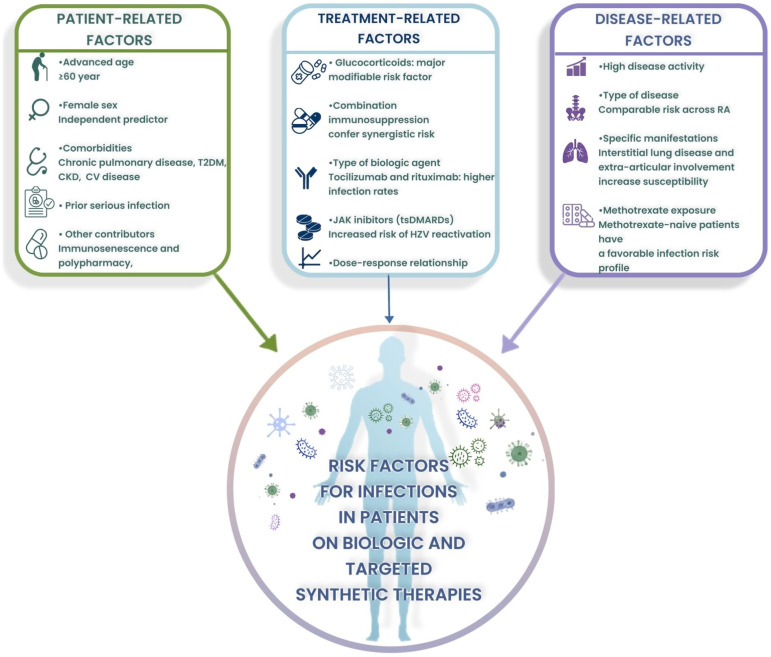
Schematic overview of risk factors for infection in patients with AD receiving biologic and targeted synthetic DMARDs. Original illustration created by the author (S. Capuccio).

In patients with ADs on bDMARDs or tsDMARDs, the risk of infection is clearly multifactorial, reflecting a complex interaction between patient characteristics, disease attributes, and treatment-related factors [128]. Understanding these risk factors is essential for suitable patient selection, risk stratification, and the implementation of effective preventive strategies aimed at reducing their clinical impact (Figure 1). Advanced age is one of the most consistent and significant risk factors. Patients with inflammatory joint diseases who start biologic treatment have a fourfold increased risk of serious infection compared with the general population, with the elevated risk remaining significant across all age groups [128].

A systematic review and meta-analysis focused specifically on older patients (≥60 years) showed that biologic users in this age group have a 2.28-fold higher risk of infection compared with younger patients (95% CI 1.57–3.31), with an overall infection prevalence of 13% versus 6%, respectively [129].

Moreover, older patients receiving biologics exhibit a 3.6-fold higher risk of infection compared with age-matched patients not on biologics (95% CI 1.62–8.01) [1,130]. The mechanisms underlying this susceptibility include immunosenescence, a higher burden of comorbidities, polypharmacy, and age-related changes in pharmacokinetics and pharmacodynamics [1,130]. Among patient-related factors, female sex is an independent predictor of both serious and non-serious infections [108]. Comorbidity burden significantly amplifies risk, with the most relevant conditions being chronic pulmonary disease, diabetes mellitus, chronic kidney disease, and cardiovascular disease. Several studies have shown that a prior history of serious infection increases by 1.62-fold the risk of a subsequent infection following initiation of biologic therapy [128,131].

Disease-related factors also play a meaningful role. Elevated disease activity is an independent risk factor, regardless of treatment. Infection risk is broadly comparable across RA, axial spondyloarthritis, and psoriatic arthritis, though certain disease manifestations, such as interstitial lung disease, confer additional susceptibility. Methotrexate-naive patients carry a more favorable infection risk profile than those with prior immunosuppressive exposure [76,108].

Treatment-related factors are equally important. Glucocorticoids represent the most significant modifiable risk factor, with a dose-dependent relationship already evident at doses ≥ 7.5 mg/day [132]. Combining biologics with corticosteroids or other immunosuppressive agents produces synergistic risk [133]. Among biologics, tocilizumab and rituximab are associated with higher infection rates than TNF inhibitors. This is due to a deeper and more specific suppression of both humoral and cellular immune responses, which impairs defense against common and opportunistic pathogens [134]. Within the TNF inhibitor class, adalimumab carries a modestly higher risk than etanercept. JAKis are notably associated with increased risk of herpes zoster reactivation. A pronounced dose–response relationship is observed, where high-dose biologics nearly double infection risk relative to standard doses [19,108].

In light of these considerations, several validated predictive models have been developed, namely RABBIT [128], DANBIO [128], and RAISE [131], to enable individualized risk estimation by integrating age, comorbidities, prior infections, biologic agent, and corticosteroid dose. These tools support shared clinical decision-making, guiding targeted preventive strategies such as vaccination and antimicrobial prophylaxis, as well as the selection of lower-risk agents in the most vulnerable patients [131].

## 6. Prevention and Clinical Recommendations 

Comprehensive prevention strategies and evidence-based clinical recommendations are indispensable to minimize infection risk in patients with autoimmune rheumatologic diseases receiving bDMARD and tsDMARD therapy.

**Figure 2:**
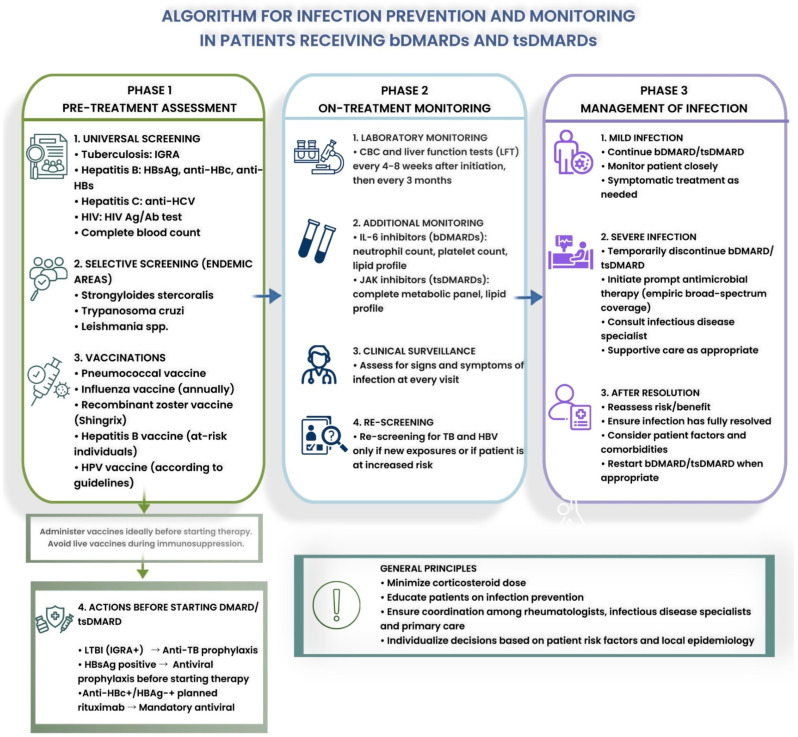
Management algorithm for infection risk assessment and prevention in patients with AD initiating bDMARDs or tsDMARDs. Abbreviations: bDMARDs, biologic disease-modifying antirheumatic drugs; tsDMARDs, targeted synthetic disease-modifying antirheumatic drugs; TB, tuberculosis; IGRA, interferon-gamma release assay; HBV, hepatitis B virus; HCV, hepatitis C virus; HIV, human immunodeficiency virus; LTBI, latent tuberculosis infection; CBC, complete blood count; JAKi, Janus kinase inhibitors; IL-6i, interleukin-6 inhibitors; VZV, varicella zoster virus. Original illustration created by the author (S. Capuccio).

A proposed management algorithm for infection prevention and monitoring in patients receiving bDMARDs and tsDMARDs is presented in Figure 2.

### 6.1. Pre-Treatment Screening

Before initiating therapy, universal screening for TB, hepatitis B and C, and HIV and complete blood count is mandatory [5,135]. For TB, the IGRA test (QuantiFERON-TB Gold) is preferred over the skin test due to its greater specificity; a positive result warrants treatment of latent TB before starting the bDMARD or tsDMARD [136]. Annual re-screening is not recommended in low-prevalence countries. For hepatitis B, HBsAg-positive patients require antiviral prophylaxis, while anti-HBc-positive, HBsAg-negative patients may be monitored, except when treated with anti-CD20 agents, where prophylaxis is mandatory [137]. Hepatitis C screening with anti-HCV antibody testing is recommended, with positive results prompting HCV RNA testing and referral for hepatology evaluation [138,139]. HIV screening should be performed universally, as undiagnosed HIV infection substantially increases opportunistic infection risk during bDMARD or tsDMARD therapy [139,140]. Parasitic screening for Strongyloides and *Trypanosoma cruzi* should be reserved for patients from endemic areas [120,123].

### 6.2. Vaccination

Vaccinations must ideally be administered before the initiation of immunosuppressive therapy. Recommended vaccines include pneumococcal (PCV15/PCV20 followed by PPSV23) [141], annual influenza, recombinant zoster vaccine (Shingrix, safe even in immunosuppressed patients), hepatitis B in at-risk individuals, and HPV according to general population guidelines [141,142]. Live vaccines are contraindicated during significant immunosuppression. In patients receiving rituximab, vaccines should be administered at least six months after the last dose [141]. For those on methotrexate, JAKis, or mycophenolate, a temporary interruption for one to two weeks after vaccination is advisable when disease activity is stable [143].

### 6.3. Antimicrobial Prophylaxis

Prophylaxis against *Pneumocystis jirovecii* pneumonia with trimethoprim–sulfamethoxazole is indicated in patients receiving ≥20 mg/day of prednisone equivalent for ≥4 weeks, particularly when combined with other immunosuppressive agents. In cases of intolerance, alternatives include dapsone, atovaquone, or aerosolized pentamidine [144].

### 6.4. On-Treatment Monitoring

Laboratory monitoring, including complete blood count and liver function tests, should be performed four to eight weeks after therapy initiation and every three months thereafter. IL-6 inhibitors additionally require monitoring of neutrophil and platelet count, and lipid profile, while JAKis necessitate a full metabolic panel. Clinical surveillance for signs and symptoms of infection should take place at every visit. Routine re-screening for TB and hepatitis B is generally not necessary but should be performed in the event of new exposures or high-risk therapies [111,145].

### 6.5. Management of Infection During Therapy

In the setting of serious infection, the most recent guidelines recommend temporary discontinuation of biologic therapy [86,124,145,146]. For minor infections, continuation with close monitoring may be appropriate. Concomitant corticosteroid doses should be tapered when feasible. Antimicrobial therapy should be initiated promptly, with broader empiric coverage considered in immunocompromised patients; infectious disease consultation is advisable in complex or severe cases [111,145].

### 6.6. Special Populations

In elderly patients (≥65 years), more aggressive preventive strategies are necessary [1,129]. During pregnancy, certolizumab is the preferred TNF inhibitor; infants exposed to biologics in utero should not receive live vaccines during the first six months of life, with the exception of rotavirus vaccine [142]. Data from the RISE registry indicate that only 15.5% of patients undergo complete screening before biologic initiation, highlighting significant implementation gaps that could be addressed through standardized protocols [140].

### 6.7. Future Directions

Optimizing the safety of bDMARD and tsDMARD therapy will depend on progress across several fronts. Standardized protocols and electronic health record reminders are needed to close the persistent gap between guidelines and everyday practice. For patients with complex profiles, particularly those from endemic regions or burdened by multiple comorbidities, close coordination among rheumatologists, infectious disease specialists, and tropical medicine experts remains indispensable [26].

Registry-based surveillance and real-world studies will continue to sharpen the understanding of long-term safety and comparative risks across agents. In this regard, validated predictive models have been developed to enable individualized risk estimation. The RABBIT Risk Score, the DANBIO infection risk score, and the recently developed RAISE prediction tool, which provides personalized 6-, 12-, 18-, and 24-month serious infection risk estimates using routinely available clinical data, represent important steps toward precision risk stratification in patients initiating advanced therapies [128,131].

Advances in personalized medicine are also emerging in the field of IBD. A recent study identified key clinical and biochemical predictors of treatment outcomes in patients with IBD, demonstrating that integrating baseline biomarkers with clinical variables can improve individualized therapeutic decision-making and further supporting the shift from a “one-size-fits-all” approach toward tailored treatment strategies [147].

Meanwhile, advances in biomarker research, including therapeutic drug monitoring, pharmacogenomics and monitoring of inflammatory markers such as fecal calprotectin and C-reactive protein, are increasingly enabling individualized treatment decisions [148,149]. The integration of multi-omics data with artificial intelligence and machine learning algorithms holds transformative potential for predicting both therapeutic response and infection susceptibility in patients receiving immunosuppressive therapy [150,151]. However, significant challenges remain, including data standardization, cost of omics profiling, interpretative complexity, and the need for prospective validation in real-world clinical settings [150]. Throughout, patient education and shared decision-making will be central to improving adherence to preventive measures and ensuring early recognition of infectious complications.

## 7. Conclusions

Biologic therapies have fundamentally transformed the therapeutic landscape of autoimmune, rheumatologic and dermatologic diseases, providing unprecedented disease control and improving quality of life for millions of patients worldwide [5,60,86]. These agents have demonstrated superior efficacy compared with conventional synthetic DMARDs in achieving clinical remission and restoring physical function in conditions such as RA, psoriatic arthritis, axial spondyloarthritis, and moderate-to-severe plaque psoriasis [152].

The expansion of therapeutic options has enabled increasingly personalized treatment approaches tailored to individual patient characteristics and disease phenotypes [71]. However, this therapeutic revolution has been accompanied by significant infectious complications that require thorough understanding, systematic prevention strategies, and careful monitoring.

The infection risk associated with bDMARD and tsDMARD therapy is multifactorial, reflecting the complex interplay between patient-related factors, disease characteristics, and treatment-specific variables [128,129]. Although the absolute increase in serious infection risk is modest, the clinical impact can be substantial, with certain opportunistic infections causing significant morbidity and mortality [76,153]. Evidence-based prevention strategies can substantially reduce infection risk while preserving the therapeutic benefits of these agents. Recent data show that only 15.5% of patients in the ACR RISE registry had documentation of appropriate pre-treatment screening for all recommended infections, highlighting a significant gap between guideline recommendations and clinical practice [140]. The benefit–risk profile remains favorable overall for most patients with autoimmune, rheumatologic and dermatologic diseases [154]. Meta-analyses demonstrate that these agents achieve clinically meaningful improvements in disease activity, physical function, and remission rates, with modest absolute increases in serious infection risk [76,152]. Notably, at least one study suggests that while bDMARDs increase the risk of hospitalization for serious infection, they may paradoxically reduce the risk of sepsis or fatal outcomes among patients who develop serious infections, likely reflecting improved overall disease control and reduced need for rescue glucocorticoids [86]. The advent of IL-23 inhibitors and other newer agents with more favorable safety profiles, combined with growing experience in dose optimization and treatment de-escalation strategies, may further improve the benefit–risk ratio [155].

In conclusion, bDMARD and tsDMARD therapies represent a major therapeutic advance for autoimmune, rheumatologic and dermatologic diseases, with well-characterized infectious risks that can be substantially mitigated through systematic screening, vaccination, antimicrobial prophylaxis when indicated, and careful monitoring. The key to optimizing outcomes lies not in avoiding these highly effective therapies but in implementing comprehensive, evidence-based prevention strategies that allow patients to benefit from disease control while minimizing infectious complications. As therapeutic options continue to expand and understanding of infection risk factors deepens, the challenge for clinicians is to translate this knowledge into consistent clinical practice, ensuring that all patients receive appropriate pre-treatment evaluation, preventive interventions, and ongoing surveillance tailored to their individual risk profile.

## Data Availability

No new data were created or analyzed in this study. Data sharing is not applicable to this article.
